# Genetic contribution of synapse-associated protein 97 to cerebellar functional connectivity changes in first-episode schizophrenia

**DOI:** 10.1186/s12888-023-05036-9

**Published:** 2023-08-29

**Authors:** Xusan Xu, Shucun Luo, Xiaoxia Wang, Xia Wen, Jingwen Yin, Xudong Luo, Bin He, Chunmei Liang, Susu Xiong, Dongjian Zhu, Dong Lv, Zhun Dai, Juda Lin, You Li, Zhixiong Lin, Wubiao Chen, Zebin Luo, Yajun Wang, Guoda Ma

**Affiliations:** 1 Institute of Neurology, Affiliated Hospital of Guangdong Medical University, Zhanjiang, 524001 China; 2 Maternal and Children’s Health Research Institute, Shunde Women and Children’s Hospital, Guangdong Medical University, Foshan, 528300 China; 3 Department of Radiology, Affiliated Hospital of Guangdong Medical University, Zhanjiang, 524001 China; 4 Institute of Neurology, Longjiang Hospital, the Third Affiliated Hospital of Guangdong Medical University, Shunde, 528300 China; 5 Department of Psychiatry, Affiliated Hospital of Guangdong Medical University, Zhanjiang, 524001 China

**Keywords:** Schizophrenia, SAP97, Rs3915512, Resting-state functional connectivity, Cerebellum

## Abstract

Our previous study data suggested that the synapse-associated protein 97 (SAP97) rs3915512 polymorphism is significantly related to clinical performance in schizophrenia. The cerebellum exhibits abundant expression of SAP97, which is involved with negative symptoms, cognition and emotion in schizophrenia. As functional dysconnectivity with the cortical-subcortical-cerebellar circuitry has been widely shown in patients with schizophrenia, cortical-subcortical-cerebellar dysconnectivity can therefore be considered a possible intermediate phenotype that connects risk genes with schizophrenia. In this study, resting-state functional magnetic resonance imaging (fMRI) was applied to evaluate whether the SAP97 rs3915512 polymorphism changes cortical/subcortical-cerebellar resting-state functional connectivity (RSFC) in 104 Han Chinese subjects (52 first-episode schizophrenia (FES) patients and 52 matched healthy controls (HCs)). To examine RSFC between cortical/subcortical regions and the cerebellum, a ROI (region of interest)-wise functional connectivity analysis was conducted. The association between abnormal cortical/subcortical-cerebellar connectivity and clinical manifestation was further assessed in FES patients with different genotypes. The interactive effect of disease and genotype on RSFC was found between the frontal gyrus (rectus) and cerebellum. A positive correlation was suggested between RSFC in the cerebellum and the hostility scores in FES patients with the A allele, and no correlation was found in FES patients with the TT genotype. The current findings identified that SAP97 may be involved in the process of mental symptoms in FES patients via cerebellar connectivity depending on the rs3915512 polymorphism genotype.

**Supplementary Information:**

The online version contains supplementary material available at 10.1186/s12888-023-05036-9.

## Introduction

Recently, a genome-wide analysis of copy-number variation (CNV) of schizophrenia identified several microdeletions in the 3q29 region, including the synapse-associated protein 97 (SAP97) gene [1]. SAP97, as a developmentally regulated gene [2], plays an important role in regulating synaptic development [3], synaptic plasticity [4] and neurotransmitter transmission [5], which is consistent with the neurodevelopmental hypothesis for the etiology of schizophrenia. Moreover, a previous study found decreased protein levels of SAP97 in postmortem brain tissues of patients with schizophrenia [6]. All of this evidence supports that SAP97 may play a pivotal role in disturbed functional connectivity in schizophrenia.

Uezato et al. identified a new splicing variant of SAP97, which is transcribed from an unreported exon (labeled exon 3b) between exons 3 and 4, and rs3915512 was reported as the only single nucleotide polymorphism (SNP) in exon 3b [7]. The T > A variation of the rs3915512 polymorphism meets the exonic splicing enhancer (ESE) consensus, resulting in the formation of an exon 3b-inserted splicing variant, and this new splicing variant might truncate the SAP97 protein because of the in-frame stop codon in exon 3b [7]. A Japanese case‒control study related the rs3915512 polymorphism to schizophrenia [8]. Moreover, our recent study revealed that the rs3915512 polymorphism showed a significant association with cognitive drawbacks in schizophrenic patients [9]. Thus, SAP97 rs3915512 may be involved in the pathophysiology of schizophrenia.

It is generally believed that schizophrenia is related to the disturbed integration of information (structural and functional connectivity) within different brain regions [10]. However, previous studies on brain connectivity in schizophrenia rarely involved the cerebellum. In addition to being traditionally associated with motor function, the cerebellum also plays a crucial role in both emotional and cognitive functions [11]. Impaired structural connectivity and functional connectivity [12] of the cerebellum in schizophrenia have also been reported. Moreover, cortical-subcortical-cerebellar circuitry dysconnectivity has been widely accepted as a convincing neuroimaging marker for schizophrenia [13]. SAP97 is dispersed throughout the brain, including the cerebral cortex [7] and cerebellum [14]. Combined with the biological roles of SAP97, we speculate that SAP97 rs3915512 may change the functional connectivity between cortical/subcortical and cerebellum and is related to some clinical characteristics. To avoid possible deviation from the disease process or treatment effect, first-episode drug-naive schizophrenic patients were recruited in the present study.

## Materials and methods

### Subjects and genotyping

This research was conducted in 104 right-handed participants, comprising 52 schizophrenic patients (30 males and 22 females, mean age = 27.29 ± 8.21 years, education years = 10.60 ± 2.70 years) and 52 healthy controls (HCs) (23 males and 29 females, mean age = 29.17 ± 8.48 years, education years = 11.62 ± 2.61 years) (Table 1). The Ethics Committee of the Affiliated Hospital of Guangdong Medical University approved this research. Informed consent was acquired from each individual prior to attending this study.

**Table 1:** Genotyping and allele distribution of SAP97 rs3915512 in HC and FES

Group	HC	FES		*P* value
n	52	52		
HWE	0.484	0.324		
Age (years)	29.17 ± 8.48	27.29 ± 8.21	*F* = 0.73	0.252
Male/female	23/29	30/22	χ2 = 1.89	0.107
Education (years)	11.62 ± 2.61	10.60 ± 2.70	*F* = 0.50	0.053
TT	30	28		
TA/AA	19/3	22/2	χ2 = 0.16	0.693

*HC* Healthy control, *FES* First episode schizophrenia, *HWE*, Hardy–Weinberg Equilibrium. These data have shown in our previous article (Xu et al., 2020)

Participants in this study came from the Health Examination Center and the Department of Psychiatry of the Affiliated Hospital of Guangdong Medical University. Every participant underwent a careful assessment by at least two senior psychiatrists in line with the diagnostic criteria of schizophrenia in the Diagnostic and Statistical Manual of Mental Disorders V (DSM-V). None of the patients had a history of substance abuse, neurological disorders, head trauma, or medical diseases involving the brain, and the HC group of this study excluded persons who had psychiatric disorders or a history of psychosis in a first-degree relative. Likewise, our study did not enroll individuals if they had magnetic resonance imaging (MRI) contraindications or other contraindications.

The Positive and Negative Syndrome Scale (PANSS) [15] was used to evaluate the severity of mental symptoms of the schizophrenic patients and consists of three subscales: positive scale (7 items), negative scale (7 items), and pathological scale (16 items). Meanwhile, the Brief Assessment of Cognition in Schizophrenia (BACS) scale [16] was conducted to explore the neurocognitive function of the patients and includes seven subscales: motor speed, semantics and verbal fluency, working memory, reasoning and problem-solving, attention and processing speed, and verbal memory.

The genomic DNA of all participants was extracted from EDTA-treated peripheral blood using the Tiangen DNA Isolation Kit (Tiangen Biotech, Beijing, China). As described before [17], the SAP97 rs3915512 polymorphism was examined using the improved multiplex ligation detection reaction (imLDR) technique (Genesky Biotech, Shanghai, China). The polymerase chain reaction (PCR) primers used for the rs3915512 polymorphism were as follows: rs3915512 forward primer: 5’-TGTTCAGGTGCATCAAGTGGTCTTTACA-3’, rs3915512 reverse primer: 5’-CTTCAGTAACTTCCAGTCAGATATGGCCT-3’. The allele-specific probes were as follows: rs3915512RA: 5’-TACGGTTATTCGGGC TCCTGTCAGTCAGATATGGCCTTACATCTATCTGTTCAT-3’, rs3915512RP: 5’-AGAATAATTGTTGGTGTGATTTGAAGACTACTTTTTTTTTTTTTTTTTTT-3’, rs3915512RT: 5’-TTCCGCGTTCGGACTGATATCAGTCAGATATG GCCTTACATCTATCTGTTCAA-3’.

### Neuroimaging acquisition

Image acquisition was performed on a 3.0 T GE Discovery MR750 scanner (GE Healthcare Systems, Milwaukee, WI, USA). During scanning, foam padding was used to minimize motion-related artifacts, and subjects were instructed to stay awake, lay still, close their eyes and clear their minds.

Resting-state functional magnetic resonance imaging (fMRI) data were acquired via an echo planar imaging (EPI) sequence (time of repetition (TR)/time of echo (TE) = 2000/30 ms; field of view (FOV) = 230 mm × 230 mm; matrix = 64 × 64; flip angle (FA) = 90°; slice thickness = 3.6 mm; slice interval = 0.6 mm; 38 scanning slices; 240 dynamics).

T1-weighted images were obtained using a three-dimensional fast field echo (FFE) pulse sequence with the following imaging parameters: TR/TE = 8.16/3.18 ms; FOV = 512 mm × 512 mm; matrix = 256 × 256; FA = 90°; slice thickness = 1 mm, slice interval = 0 mm; 172 scanning slices.

### Data processing

The data processing assistant for resting-state fMRI (DPARSF_V5.3, Cognitive and Brain Diseases Centre of the Hangzhou Normal University) software, running on MATLAB 2012a, was applied to analyze the fMRI data. The detailed steps for the fMRI data processing included the following: (1) convert format: the DICOM format was converted to NIfTI format; (2) remove the first 10 slices for each scan; (3) slice timing correction; (4) correct rigid-body head motion: rotational or translational motion parameters less than 2.5° or 2.5 mm; (5) normalize to the MNI template space (resampled with voxels of 3 mm × 3 mm × 3 mm); (6) spatial smoothing: 6 mm full width at half maximum (FWHM) Gaussian kernel; (7) filtering 0.01–0.08 Hz band; (8) nuisance signal regression: head motion parameters were performed by using the Friston 24 model, global signal, cerebrospinal fluid signal and white matter signal; (9) define the region of interest (ROI): the Anatomical Automatic Labeling (AAL) atlas was selected (the Montreal Neurological Institute (MNI) coordinates of the 26 cerebellar regions used in this study are shown in Supplementary information Table S1); (10) compute functional connectivity maps, and conduct Fisher r-to-z transformation using DPARSF. Finally, the resting-state functional connectivity (RSFC) value was obtained from these z-maps.

### Statistical analyses

Data from the demographic and scales were analyzed using SPSS 21.0 software. The dominant model (TA + AA vs. TT) was applied in this research. Pearson’s chi-square test was conducted for categorical variables, and Student’s t tests were performed for continuous variables. The significance level was set at *P* < 0.05 with two-tailed tests. To evaluate the interactive effect of disease and genotype on RSFC, age, educational year, and sex as covariates, 2 × 2 analysis of covariance (ANCOVA) was used. After Bonferroni correction, *P* < 1.92E-03 was considered to indicate a significant difference. The post hoc t test analysis was further performed. *P* < 0.0125 was considered statistically significant using Bonferroni correction.

### Correlation between clinical symptoms and altered functional connectivity

We examined the Spearman correlation between functional connectivity and clinical performance (positive, negative and general pathopsychological symptom scores evaluated via the PANSS and cognition assessed using the BACS). Correction for multiple comparisons was conducted using Bonferroni correction, and *P* < 0.033 for PANSS or *P* < 7.14E-03 for BACS was defined to indicate a significant difference.

## Results

Fifty-two FES patients and 52 HCs of Chinese Han ethnicity with demographic, clinical performance score, genotype and fMRI imaging data were included in this study. The genotype distribution of SAP97 rs3915512 was in accordance with Hardy Weinberg equilibrium (*P* > 0.05). No significant difference (*P* > 0.05) was found in age, educational year, sex, genotype distribution, PANSS scores and BACS scores between the FES and HC groups (Tables 1 and 2).

**Table 2:** Clinical scales of the schizophrenic patients and distribution by genotypes of the rs3915512 polymorphism

Group	FES		*P* value
PANSS	TT(*n* = 26)	TA + AA(*n* = 24)	
Total score	80.62 ± 23.24	78.67 ± 18.40	0.745
Positive score	25.54 ± 9.24	22.29 ± 9.34	0.223
Negative score	17.62 ± 13.16	20.83 ± 14.72	0.418
Pathological score	37.46 ± 10.98	35.54 ± 8.22	0.490
BACS	TT(*n* = 17)	TA + AA(*n* = 15)	
Working memory	23.47 ± 5.04	19.38 ± 6.28	0.067
Semantics fluency	33.77 ± 9.89	30.92 ± 6.16	0.393
Letter fluency	12.33 ± 5.23	12.08 ± 2.97	0.884
Verbal memory	37.27 ± 8.75	31.78 ± 8.79	0.180
Motor speed	45.35 ± 14.82	43.10 ± 14.06	0.701
Reasoning and problem solving	15.58 ± 5.18	12.64 ± 4.90	0.177
Attention and processing speed	33.46 ± 9.79	32.58 ± 9.37	0.821

*FES* First episode schizophrenia, *PANSS* Positive and Negative Syndrome Scale, *BACS* Brief Assessment of Cognition in Schizophrenia. Part of the data (BACS) have been reported in our previous article. (Xu et al., 2020)

**Fig. 1:**
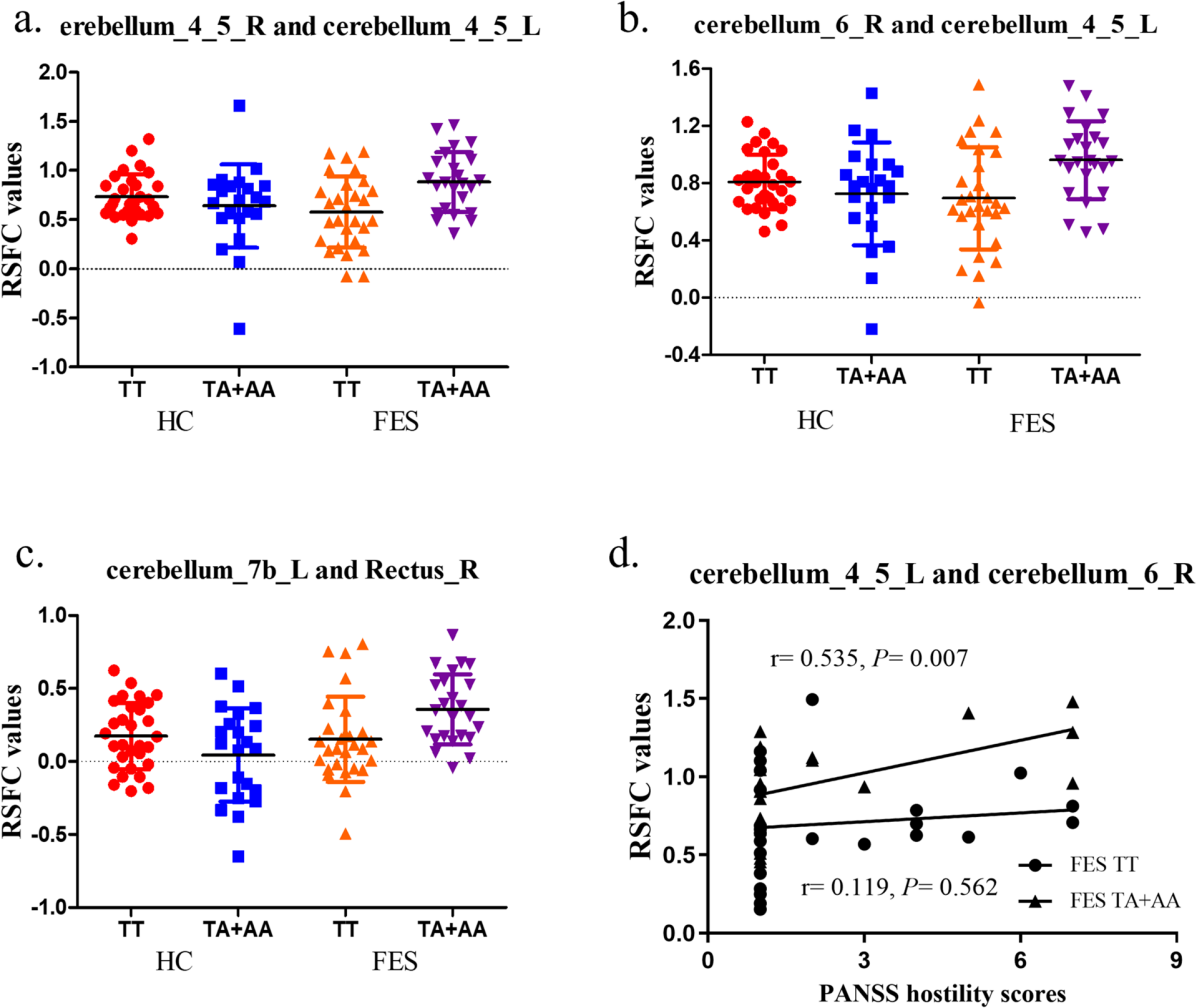
**a**-**c**, post hoc analysis. Post hoc analysis of the interactive effect between cerebellum_4_5_R and cerebellum_4_5_L (**a**), cerebellum_6_R and cerebellum_4_5_L (**b**), cerebellum_7b_L and Rectus_R (**c**); R: right; L: left; RSFC: resting state functional connectivity (Bonferroni correction, *P* < 0.0125). **d** Correlation analysis of PANSS hostility scores and RSFC between the left cerebellum_4_5 and right cerebellum_6, FES: first episode schizophrenia; RSFC: resting state functional connectivity; PANSS: the Positive and Negative Symptom Scale; R: right; L: left

A significant interactive effect in RSFC of the right rectus was found in the left cerebellum_7b (*P* = 1.90E-03), and RSFC of the left cerebellum_4_5 was observed in the right cerebellum_4_5 and right cerebellum_6 (*P* = 1.65E-03 and 1.70E-03, respectively) (Table 3). Post hoc t test analysis showed that the patients with the A allele had higher RSFC values than the patients with the TT genotype and the control group with the A allele (*P* < 0.05) (Table 3 and Fig. 1 a-c).

**Table 3:** Interactive effect and post hoc analysis of RSFC values between SAP97 rs3915512 genotype and disease

		RSFC value						post hoc analysis of genotype in FES		post hoc analysis of genotype in HC		post hoc analysis of diagnosis in TT		post hoc analysis of diagnosis in TA + AA	
AAL		HC		FES		Interactive effect									
		TT	TA + AA	TT	TA + AA	*F*	*P*	*F*	*P*	*F*	*P*	*F*	*P*	*F*	*P*
cerebelum_4_5_R	cerebelum_4_5_L	0.74 ± 0.22	0.64 ± 0.42	0.58 ± 0.36	0.88 ± 0.30	10.488	**1.65E-03**	11.878	**8.41E-04**	1.348	0.249	4.620	0.034	5.531	0.021
cerebelum_6_R	cerebelum_4_5_L	0.81 ± 0.19	0.73 ± 0.36	0.70 ± 0.36	0.96 ± 0.27	10.421	**1.70E-03**	11.787	**8.79E-04**	1.344	0.249	3.862	0.052	6.290	0.014
cerebelum_7b_L	Rectus_R	0.17 ± 0.23	0.04 ± 0.32	0.15 ± 0.29	0.36 ± 0.24	10.194	**1.90E-03**	7.864	**0.006**	3.020	0.085	0.222	0.639	14.734	**2.21E-04**

*AAL* Anatomical Automatic Labeling, *HC* Healthy control, *FES* First episode schizophrenia, *R* Right, *L* Left

Values are the mean ± SD; 2 × 2 ANCOVA *P* < 1.92E-03 (Bonferroni correction); The bold values in the post hoc analysis can survive for Bonferroni correction (*P* < 0.0125)

The Spearman correlation revealed a significant positive correlation between RSFC of the left cerebellum_4_5 and right cerebellum_6 and PANSS hostility scores (*r* = 0.535, *P* = 0.007) in FES patients with the A allele but not in FES patients with the TT genotype (*r* = 0.119, *P* = 0.562) (Fig. 1 d).

## Discussion

The cerebellum is thought to be involved in the coordination or modulation of a range of cognitive and emotional processes of cortical activity through the frontal-thalamic-cerebellar circuitry in schizophrenia [18]. Recent studies have reported reduced volume [19], decreased gray matter density [20] of the cerebellum, and disturbed functional connectivity between the cerebellum and frontal lobe [12] in schizophrenia. In this imaging genetic analysis, our data revealed a significant genotype × disease interactive effect in the cerebellum or between the cerebellum and frontal gyrus (rectus) but found no traditional thalamic-cerebellar dysconnectivity in FES patients. The SAP97 rs3915512 TT genotype showed lower functional connectivity than A allele carriers in the FES group. Moreover, higher functional connectivity may predict more serious hostility performance in patients with the A allele but not in FES patients with the TT genotype. These findings suggested that SAP97 influences cerebellar coupling and its association with psychological obstacles in schizophrenia.

One of the main findings of this research was the impact of SAP97 rs3915512 on cerebellar functional connectivity. SAP97 is expressed in both the frontal lobe and cerebellum, and decreased mRNA expression has been reported in the prefrontal lobe in patients with schizophrenia [7]. The essential role of SAP97 in regulating long-term potentiation [4], glutamate transport [5], synaptic development and plasticity [3] was reported. Therefore, we speculate that SAP97 rs3915512 may affect the development and plasticity of the frontal-cerebellar circuitry by influencing frontal-cerebellar connectivity. Consistent with our previous case‒control study (1138 patients with schizophrenia vs. 1036 healthy controls) [9, 21], no relationship was found between the rs3915512 polymorphism and patient diagnosis, which suggested that the effects of SAP97 in schizophrenia may not confer risk for the disorder. However, this study is different because it also failed to relate the rs3915512 polymorphism to clinical performance. We think this may be because of (1) the limited sample size and (2) the inclusion of first-episode patients with mild symptoms. Moreover, fMRI found altered functional and structural connectivity even in early schizophrenia [22]. Thus, combining fMRI and risk gene detection may help early identification and treatment in schizophrenic patients.

A more interesting result of this study was that individual cerebellar functional connectivity affected hostility scores in a completely different pattern depending on the SAP97 rs3915512 genotype. Considering the wide hypoconnectivity within cortical-subcortical-cerebellar regions in schizophrenia [23] and the evidence that SAP97 can enhance AMPAR responses in the low activity state of prefrontal neurons [24], we speculate that the change in SAP97 protein structure caused by the T > A variation in the rs3915512 polymorphism [7] with higher RSFC fails to have such an enhanced function [25]. Thus, schizophrenic patients with the rs3915512 A allele showed more severe mental symptoms.

There are some shortcomings in this study. First, because there is no uniform cerebellar template, the selection of seed regions is mainly based on the AAL template, which may not completely exclude white matter. Thus, it is essential to replicate the findings with a larger sample size to confirm the accuracy of the conclusions. Finally, the precise mechanism of the SAP97 gene in the neurobiology of schizophrenia remains unclear and needs further study.

## Conclusions

In summary, our data revealed that SAP97 rs3915512 may play a crucial role in cerebellar dysconnectivity in schizophrenia. SAP97 plays a distinct role in regulating the hostility performance of first-episode schizophrenia patients via the cerebellar circuitry depending on the rs3915512 genotype. Considering the biological heterogeneity of schizophrenia, it is essential to replicate the findings of this study using multicenter research.

### Supplementary Information

**Additional file 1: Table S1.** 26 cerebellar regions extracted from the AAL template.

## Data Availability

The datasets generated and analyzed during the current study are available in the dbSNP repository, dbSNP accession: ss2137544405, (dbSNP Build ID: B151). Available from: https://www.ncbi.nlm.nih.gov/SNP/snp_viewTable.cgi?handle=NEUROLOGY_GDMU.

## References

[1] Mulle JG, Dodd AF, McGrath JA, Wolyniec PS, Mitchell AA, Shetty AC, Sobreira NL, Valle D, Rudd MK, Satten G (2010). Microdeletions of 3q29 confer high risk for schizophrenia. Am J Hum Genet.

[2] Hiraoka S, Kajii Y, Kuroda Y, Umino A, Nishikawa T (2010). The development- and phencyclidine-regulated induction of synapse-associated protein-97 gene in the rat neocortex. Eur Neuropsychopharmacol.

[3] Yang X, Gong R, Qin L, Bao Y, Fu Y, Gao S, Yang H, Ni J, Yuan TF, Lu W (2022). Trafficking of NMDA receptors is essential for hippocampal synaptic plasticity and memory consolidation. Cell Rep.

[4] Howard MA, Elias GM, Elias LA, Swat W, Nicoll RA (2010). The role of SAP97 in synaptic glutamate receptor dynamics. Proc Natl Acad Sci U S A.

[5] Kay Y, Tsan L, Davis EA, Tian C, Decarie-Spain L, Sadybekov A, Pushkin AN, Katritch V, Kanoski SE, Herring BE (2022). Schizophrenia-associated SAP97 mutations increase glutamatergic synapse strength in the dentate gyrus and impair contextual episodic memory in rats. Nat Commun.

[6] Toyooka K, Iritani S, Makifuchi T, Shirakawa O, Kitamura N, Maeda K, Nakamura R, Niizato K, Watanabe M, Kakita A (2002). Selective reduction of a PDZ protein, SAP-97, in the prefrontal cortex of patients with chronic schizophrenia. J Neurochem.

[7] Uezato A, Yamamoto N, Iwayama Y, Hiraoka S, Hiraaki E, Umino A, Haramo E, Umino M, Yoshikawa T, Nishikawa T (2015). Reduced cortical expression of a newly identified splicing variant of the DLG1 gene in patients with early-onset schizophrenia. Transl Psychiatry.

[8] Uezato A, Yamamoto N, Jitoku D, Haramo E, Hiraaki E, Iwayama Y, Toyota T, Umino M, Umino A, Iwata Y (2017). Genetic and molecular risk factors within the newly identified primate-specific exon of the SAP97/DLG1 gene in the 3q29 schizophrenia-associated locus. Am J Med Genet B Neuropsychiatr Genet.

[9] Xu X, Liang C, Lv D, Yin J, Luo X, Fu J, Yan H, Zhou X, Dai Z, Zhu D (2018). Association of the Synapse-Associated Protein 97 (SAP97) gene polymorphism with neurocognitive function in schizophrenic patients. Front Psychiatry.

[10] Guo JY, Ragland JD, Carter CS (2019). Memory and cognition in schizophrenia. Mol Psychiatry.

[11] Schmahmann JD (2019). The cerebellum and cognition. Neurosci Lett.

[12] Zhuo C, Wang C, Wang L, Guo X, Xu Q, Liu Y, Zhu J (2018). Altered resting-state functional connectivity of the cerebellum in schizophrenia. Brain Imaging Behav.

[13] Brady RO, Gonsalvez I, Lee I, Ongur D, Seidman LJ, Schmahmann JD, Eack SM, Keshavan MS, Pascual-Leone A, Halko MA (2019). Cerebellar-Prefrontal network connectivity and negative symptoms in schizophrenia. Am J Psychiatry.

[14] Leonoudakis D, Conti LR, Radeke CM, McGuire LM, Vandenberg CA (2004). A multiprotein trafficking complex composed of SAP97, CASK, Veli, and Mint1 is associated with inward rectifier Kir2 potassium channels. J Biol Chem.

[15] Nicotra E, Casu G, Piras S, Marchese G (2015). On the use of the positive and negative syndrome Scale in randomized clinical trials. Schizophr Res.

[16] Poznanovic ST, Markovic M, Stasevic M, Karlicic IS, Tomanic M: Cross-Cultural Adaptation and Validation of the Serbian Version of the Brief Assessment of Cognition in Schizophrenia Scale. Int J Environ Res Public Health. 2023;20(4):3699. 10.3390/ijerph20043699 PMC9959884 36834393

[17] Xu X, He B, Lin Z, Wang X, Yin J, Luo X, Luo S, Liang C, Wen X, Xiong S (2020). SAP97 rs3915512 polymorphism affects the neurocognition of schizophrenic patients: a genetic neuroimaging study. Front Genet.

[18] Andreasen NC, Pierson R (2008). The role of the cerebellum in schizophrenia. Biol Psychiatry.

[19] Moussa-Tooks AB, Rogers BP, Huang AS, Sheffield JM, Heckers S, Woodward ND (2022). Cerebellar structure and cognitive ability in psychosis. Biol Psychiatry.

[20] Wang J, Zhou L, Cui C, Liu Z, Lu J (2017). Gray matter morphological anomalies in the cerebellar vermis in first-episode schizophrenia patients with cognitive deficits. BMC Psychiatry.

[21] Xu X, Wang Y, Zhou X, Yin J, Yu H, Wen X, Lv D, Zhu D, Xiong S, Yan H (2020). The genetic variations in SAP97 gene and the risk of schizophrenia in the Chinese Han population: a further study. Psychiatr Genet.

[22] Lahti AC (2023). Discovery of early schizophrenia through neuroimaging. Psychiatry Res.

[23] Ferri J, Ford JM, Roach BJ, Turner JA, van Erp TG, Voyvodic J, Preda A, Belger A, Bustillo J, O'Leary D (2018). Resting-state thalamic dysconnectivity in schizophrenia and relationships with symptoms. Psychol Med.

[24] Yuen EY, Yan Z (2011). Cellular mechanisms for dopamine D4 receptor-induced homeostatic regulation of alpha-amino-3-hydroxy-5-methyl-4-isoxazolepropionic acid (AMPA) receptors. J Biol Chem.

[25] Schluter OM, Xu W, Malenka RC (2006). Alternative N-terminal domains of PSD-95 and SAP97 govern activity-dependent regulation of synaptic AMPA receptor function. Neuron.

