# Supplementary material for: Genetic contribution of synapse-associated protein 97 to cerebellar functional connectivity changes in first-episode schizophrenia
Source: BMC Psychiatry. 2023 Aug 29;23:630. doi: 10.1186/s12888-023-05036-9 (PMC10464201; doi:10.1186/s12888-023-05036-9)
Supplement: Supplementary file 1 — Additional file 1: Table S1. 26 cerebellar regions extracted from the AAL template. [file 12888_2023_5036_MOESM1_ESM.doc]

**Genetic contribution of synapse-associated protein 97 in cerebellum functional connectivity changes in the first episode schizophrenia**

Xusan Xu^1, 2#^, Shucun Luo^3#^, Xiaoxia Wang^1, 4#^, Xia Wen^1^, Jingwen Yin^5^, Xudong Luo^5^, Bin He^5^ Chunmei Liang^1^, Susu Xiong^5^, Dongjian Zhu^5^, Dong Lv^5^, Zhun Dai^5^, Juda Lin^5^, You Li^1^, Zhixiong Lin^5^, Wubiao Chen^3^, Zebin Luo^3*^, Yajun Wang^2*^, Guoda Ma^1,2*^

1. Institute of Neurology, Affiliated Hospital of Guangdong Medical University, Zhanjiang, 524001, China

2. Maternal and Children's Health Research Institute, Shunde Women and Children’s Hospital, Guangdong Medical University, Foshan, 528300, China

3. Department of Radiology, Affiliated Hospital of Guangdong Medical University, Zhanjiang, 524001, China

4. Institute of Neurology, Longjiang Hospital, the Third Affiliated Hospital of Guangdong Medical University, Shunde, 528300, China

5. Department of Psychiatry, Affiliated Hospital of Guangdong Medical University, Zhanjiang, 524001, China

| Table S1. 26 cerebellar regions extracted from the AAL template. | | | |
| --- | --- | --- | --- |
| AAL | MNI | AAL | MNI |
| Vemis_1_2 | 1,-41,-23 | cerebelum_6_R | 8,-70,-24 |
| Vemis_3 | 1,-40,-17 | cerebelum_7b_L | -8,-72,-41 |
| Vemis_4_5 | 2,-52,-16 | cerebelum_7b_R | 21,-75,-46 |
| Vemis_6 | 1,-65,-18 | cerebelum_8_L | -33,-63,-45 |
| Vemis_7 | 2,-64,-26 | cerebelum_8_R | 30,-70,-50 |
| Vemis_8 | 0,-66,-34 | cerebelum_9_L | -14,-49,-43 |
| Vemis_9 | 0,-52,-44 | cerebelum_9_R | 11,-50,-43 |
| Vemis_10 | 0,-48,-33 | cerebelum_10_L | -25,-30,-41 |
| cerebelum_3_L | -10,-33,-17 | cerebelum_10_R | 26,-33,-43 |
| cerebelum_3_R | 16,-30,-21 | cerebelum_Crus1_L | -35,-68,-29 |
| cerebelum_4_5_L | -28,-31,-31 | cerebelum_Crus1_R | 30,-68,-30 |
| cerebelum_4_5_R | 27,-31,-29 | cerebelum_Crus2_L | -8,-74,-33 |
| cerebelum_6_L | -38,-56,-22 | cerebelum_Crus2_R | 10,-80,-28 |
| MNI: Montreal Neurological Institute; AAL: Anatomical Automatic Labeling, R: right; L: left. | | | |
